# Protective Effect of Ginseng Polysaccharides on Influenza Viral Infection

**DOI:** 10.1371/journal.pone.0033678

**Published:** 2012-03-19

**Authors:** Dae-Goon Yoo, Min-Chul Kim, Min-Kyung Park, Kyoung-Mi Park, Fu-Shi Quan, Jae-Min Song, Jae Joon Wee, Bao-Zhong Wang, Young-Keol Cho, Richard W. Compans, Sang-Moo Kang

**Affiliations:** 1 Department of Microbiology and Immunology, Emory University School of Medicine, Atlanta, Georgia, United States of America; 2 Department of Infectious Disease, College of Veterinary Medicine, University of Georgia, Athens, Georgia, United States of America; 3 Center for Inflammation, Immunity, and Infection, and Department of Biology, Georgia State University, Atlanta, Georgia, United States of America; 4 Department of Human Nutrition and Food Science, Chungwoon University, Namjang-Ri, Hongsung-Eup, Hongsung-Kun, Chungnam, Korea; 5 Department of Medical Zoology, Kyung Hee University School of Medicine, Seoul, Korea; 6 Department of Global Medical Science, Sungshin Women's University, Seoul, Korea; 7 Research and Development Institute, Korea Ginseng Corporation, Taejeon, Korea; 8 Department of Microbiology, University of Ulsan College of Medicine, Asan Medical Center, Seoul, Korea; University of Hong Kong, Hong Kong

## Abstract

Ginseng polysaccharide has been known to have multiple immunomodulatory effects. In this study, we investigated whether *Panax ginseng* polysaccharide (GP) would have a preventive effect on influenza infection. Administration of mice with GP prior to infection was found to confer a survival benefit against infection with H1N1 (A/PR/8/34) and H3N2 (A/Philippines/82) influenza viruses. Mice infected with the 2009 H1N1 virus suspended in GP solution showed moderately enhanced survival rates and lower levels of lung viral titers and the inflammatory cytokine (IL-6). Daily treatment of vaccinated mice with GP improved their survival against heterosubtypic lethal challenge. This study demonstrates the first evidence that GP can be used as a remedy against influenza viral infection.

## Introduction

Influenza is a serious respiratory disease causing recurrent outbreaks significantly affecting human health, livestock, and the global economy. Diverse influenza A viruses with different combinations of hemagglutinin (H1 to H16) and neuraminidase (N1 to N9) subtypes have been identified [1]. The new 2009 H1N1 influenza virus spread rapidly to over 74 countries around the world by early June 2009, when the World Health Organization raised the global outbreak alert level to the pandemic phase. [2]–[5]. Current vaccines are only effective if they are well matched with the predicted influenza strains that circulate during the next season. In addition, a number of influenza variants have evolved to develop resistance to antiviral drugs [6]. A preventive measure that would have protective effects on influenza virus regardless of strain is highly desirable.

An acidic polysaccharide from *Panax ginseng* (ginseng polysaccharide) has been shown to display immunomodulatory effects either in an immuno-stimulatory or in an immuno-suppressive manner depending on timing of treatments and disease environments. Ginseng polysaccharide (GP) promoted the production of cytotoxic cells against tumors and stimulated macrophages to produce helper types 1 and 2 (Th1 and Th2) cytokines [7]–[14]. GP was also shown to modulate the antioxidant defense systems such as superoxide dismutase and glutathione peroxidase enzymes probably via inducing regulatory cytokines [9], [15]. As an anti-inflammatory function, recent studies reported that pretreatment with GP suppressed acute inflammatory responses at an early phase resulting in the enhancement of antimicrobial activities and protection of mice from *Staphylococcus aureus*-induced sepsis [16], [17]. However, the effects of GP on viral infection remain unknown.

In this study, we have investigated the potential effects of GP on survival outcomes after influenza viral infection in a mouse model. Also, the potential roles of GP in conferring protection to naïve mice and in improving cross-protection of vaccinated mice have been explored and discussed.

## Materials and Methods

### Cells, virus, and reagents

**Figure 1:**
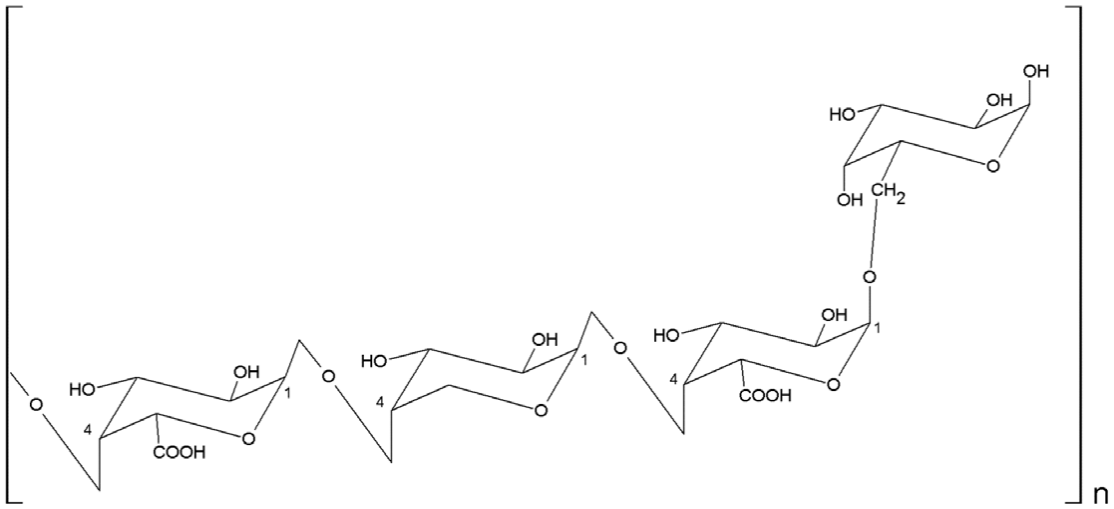
A proposed chemical structure of active substance of red ginseng acidic polysaccharides (GP). A proposed partial structure of polysaccharides present in GP was suggested in a previous study [20]. A proposed active component of GP comprises a galactogalacturonan core such as α-D-Galacturonic acid-(1-4)-α-D- Galacturonic acid-(1-4)-α-D- Galacturonic acid-(1-6)-α-D-Galactose as the acidic moiety [20].

*Spodoptera frugiperda* sf9 insect cells purchased from the American Type Culture Collection (ATCC, CRL-1711) were used for the production of recombinant baculoviruses (rBVs) and virus-like particles (VLPs) as an influenza vaccine candidate, and were cultured in SF900-II serum-free medium (GIBCO-BRL) at 27°C in spinner flasks. The influenza subtype H1N1 A/PR/8/34 (A/PR8) and H3N2 A/Philippines/82 viruses were kindly provided by Dr. Huan Nguyen, and the new 2009 H1N1 influenza virus (A/California/04/2009) by Dr. Richard Webby. Influenza viruses were grown in 11-day old embryonated hen's eggs. Egg allantoic fluids were harvested and stored at −80°C until use. Madin-Darby canine kidney (MDCK) cells purchased from ATCC were maintained in Dulbecco's modified Eagle's medium (DMEM) and used to determine virus titers from egg allantoic fluids and mouse lung homogenates by a plaque assay [18]. Mice were infected with serial dilutions of influenza virus and the 50% lethal dose (LD_50_) was determined. The ginseng polysaccharide (GP) reagent used in this study was obtained from the Korea Ginseng Corporation. Briefly, red ginseng (*P. ginseng*) was percolated with 5 volumes of 85% ethanol to extract off ethanol-soluble materials. The remaining residues were re-percolated with 5 volumes of distilled water, and the water-soluble extracts were concentrated with a vacuum evaporator. The concentrate was dialyzed against water to completely cut off small molecules of less than 15 kDa. Four volumes of absolute ethanol were added to precipitate the polysaccharide in the inner dialysate. The precipitate was dried in a vacuum drying oven, and was finally used as a red ginseng acidic polysaccharide (GP) as previously described [11], [19], [20]. An active acidic polysaccharide moiety (Table 1, Fig. 1) present in GP was reported to be composed of a galactogalacturonan core such as α-D-Galacturonic acid-(1-4)-α-D-Galacturonic acid-(1-4)-α-D-Galacturonic acid-(1-6)-α-D-Galactose [20]. GP is also known to contain sugars composed of α(1-6) glucopyranoside and β(2-6) fructofuranoside [11], [16]. As a quality control, the endotoxin content in GP preparations was less than the threshold level (0.006 EU/mg) as determined by the *Limulus amebocytes* lysates assay (EndosafeJ, Charles River Laboratories, USA) according to the manufacturer's instructions [16], [20], [21].

**Table 1:** The composition of red ginseng acidic polysaccharide (GP)*.

Composition	Molar ratios (%)
Glucuronic acid (acidic)	51.8
Galacturonic acid (acidic)	5.1
Glucose (Neutral)	26.1
Arabinose (Neutral)	1.6
Protein	<0.1

* GP has heterogeneous molecular weights with a range of 12 KDa to 450 KDa. [20].

### Ginseng polysaccharide pretreatment of mice and influenza virus infection

A powder form of GP was dissolved in phosphate-buffered saline (PBS, pH 7.4), filtered through 0.4 µm Millipore membranes. For animal experiments, 8–10 weeks old BALB/c mice (Harlan Laboratories, Indianapolis, IN) were lightly anesthetized with isoflurane inhalation and then GP was administered intranasally or intravenously in a dose ranging from 12.5 to 50 mg/kg weight. To determine the effects of GP treatment on influenza infection, mice (n = 6 to 10 per group) were anesthetized by isoflurane inhalation and intranasally infected by mouse-adapted pathogenic strains of A/PR8 or A/Philippines/82. Mice were monitored daily to record weight changes and mortality. All animal experiments and husbandry involved in the studies presented in this manuscript were conducted under the guidelines of the Emory University Institutional Animal Care and Use Committee (IACUC). Emory IACUC operates under the federal Animal Welfare Law (administered by the USDA) and regulations of the Department of Health and Human Services. Emory University IACUC specifically approved full details of this study as proposed by the approved IACUC DAR-2000161-081313.

### Lung viral titers and cytokine assays

Lung viral titers were performed using MDCK cells as previously described [22]. Lungs were collected at day 4 post infection. Briefly, serially diluted lung extracts were added to six-well plates containing confluent MDCK cell monolayers, and incubated at 37°C for 1 h. Overlay medium containing DEAE dextran, nonessential amino acids, glutamine, and trypsin were added and incubated for 2 or 3 days. After fixing with 0.25% glutaraldehyde and staining with 1% crystal violet, the plaques were counted. Cytokine ELISA was performed as described previously [23]. Ready-Set-Go interferon (IFN) γ and interleukin (IL)-6)- kits (eBioscience, San Diego, CA) were used for detecting cytokine levels in lung extracts following the manufacturer's recommended procedures.

### *In vivo* infection with mixtures of ginseng polysaccharide and virus

Different concentrations of ginseng polysaccharide (25 µl) or PBS buffer control were mixed with influenza virus (25 µl with 3 LD_50_) and incubated at room temperature for 30 min. The virus and ginseng polysaccharide mixtures were administered to naïve mice, and body weight changes and survival rates were monitored daily.

### Treatment of vaccinated mice with GP and heterosubtypic challenge

To vaccinate mice, influenza VLPs containing hemagglutinin (HA) and matrix (M1) proteins from H1N1 A/PR8 virus were prepared as described previously [18]. Mice were intramuscularly vaccinated with influenza VLPs (8 µg total protein per mouse) one time followed by daily administration with GP (0.1 mg) intranasally for 12 days from the day 1 post vaccination. On day 13 post vaccination, vaccinated mice were challenged with a lethal dose of H3N2 heterosubtypic virus (A/Philippines/82, 6 LD_50_). Body weight changes and survival rates were monitored daily.

### Antibody determination and hemagglutination activity assay

Influenza virus-specific total IgG antibodies were determined using standard methods as described previously [18], [24]. Briefly, inactivated influenza virus was used as an antigen on 96 well microtiter plates (Nunc, Rochester, NY) in coating buffer (0.1 M sodium carbonate, pH 9.5). Serially diluted serum samples were added and incubated for 1 hr at 37°C, and then horseradish peroxidase (HRP) -conjugated goat anti-mouse IgG antibody was used as secondary antibody and O-phenylenediamine as a substrate. Antibody levels are presented based on optical spectrophotometer readings at 450 nm (OD_450_).

For determination of hemagglutination activity as a measure of viral growth, virus culture supernatants were diluted, mixed with 0.5% chicken red blood cells, and then incubated for 1 hour at room temperature. The dilutions showing a blood cell precipitated spot were used as an endpoint as described previously [18], [24].

### Statistical analysis

To determine the statistical significance, a two-tailed Student's t-test was used when comparing two different groups. A *p* value less than 0.05 was considered to be significant.

## Results

### Ginseng polysaccharide protects mice from lethal infection with H1N1 influenza virus

**Figure 2:**
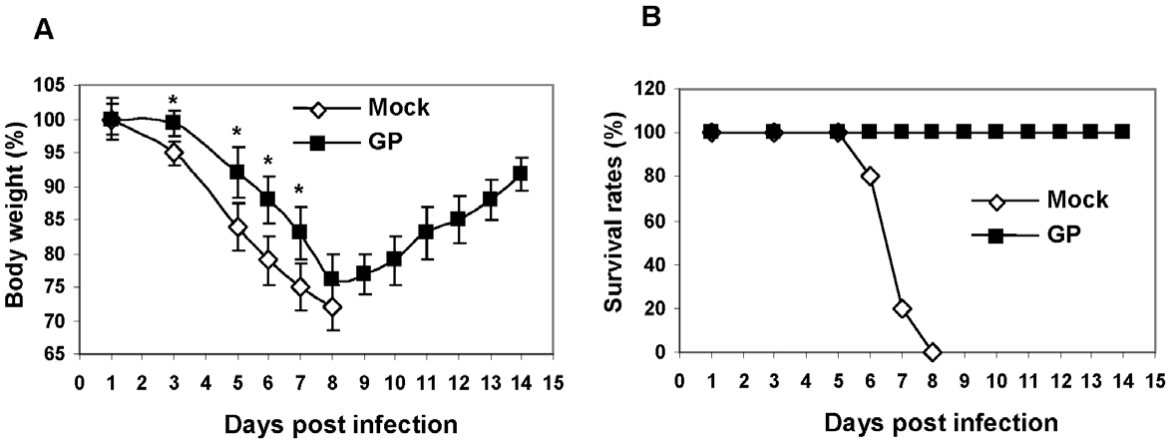
Ginseng polysaccharide pretreatment provided protection against lethal infection with H1N1 influenza virus. A) Changes in body weight (%). B) Survival rates (%). BALB/c mice (n = 10, 12 weeks old) were pretreated once intranasally with ginseng polysaccharide (25 mg/kg weight) 6 hours prior to infection with 150 plaque-forming units (pfu) of A/PR/8/34 virus (3 LD_50_). Mock: mice treated with PBS, GP: mice treated ginseng polysaccharide. * p<0.05 between mock and GP groups as determined by Student's t-test.

We have investigated the effects of ginseng polysaccharide (GP) on conferring protection against lethal infection with influenza virus in a mouse model. Mice (BALB/c) were intranasally administered 25 mg GP/kg body weight. At six hours after treatment with GP, the treated or naïve mice were intranasally infected with a lethal dose (3 LD_50_) of influenza H1N1 virus A/PR/8/34 (LD_50_: 50% lethal dose) and monitored daily for morbidity (body weight changes) and mortality (survival rates). As shown in Fig. 2A, naïve (untreated) mice showed severe body weight loss over 25% and died or were euthanized by day 8 post infection with A/PR/8/34. GP treated mice exhibited a significant delay in body weight loss although these mice also showed approximately 20–25% loss in body weight (Fig. 2A). Importantly, the GP treated mice all survived lethal infection (Fig. 2B). When mice were treated with GP at an earlier time point 24-hr before infection, 30% of GP treated mice were protected while none of the naïve control group survived challenge infection with A/PR/8/34 (3 LD_50_).

To determine the dosage effects of GP, different doses of GP were similarly tested in mice with the same challenge dose (3 LD_50_) as summarized in the Table 2. The lowest dose of GP (12.5 mg/kg) provided only partial protection of 60% of the treated mice indicating less protective efficacy (Table 2). The higher dose (50 mg GP/kg) provided 80–100% protection following severe body weight loss, which indicates no improved protection with the higher dose of GP.

**Table 2:** Dosage effects of GP on protection against influenza virus (A/PR/8/34)*.

GP dosage (mg GP/Kg)	Survival (%)	Weight loss (%)
0	0	>25
12.5	60	20–25
25	100	20–25
50	90–100	20–25

* Each group has 5–10 mice. GP was intranasally administered to mice with the given dose.

### Ginseng polysaccharide confers protection against lethal infection with H3N2 influenza virus

**Figure 3:**
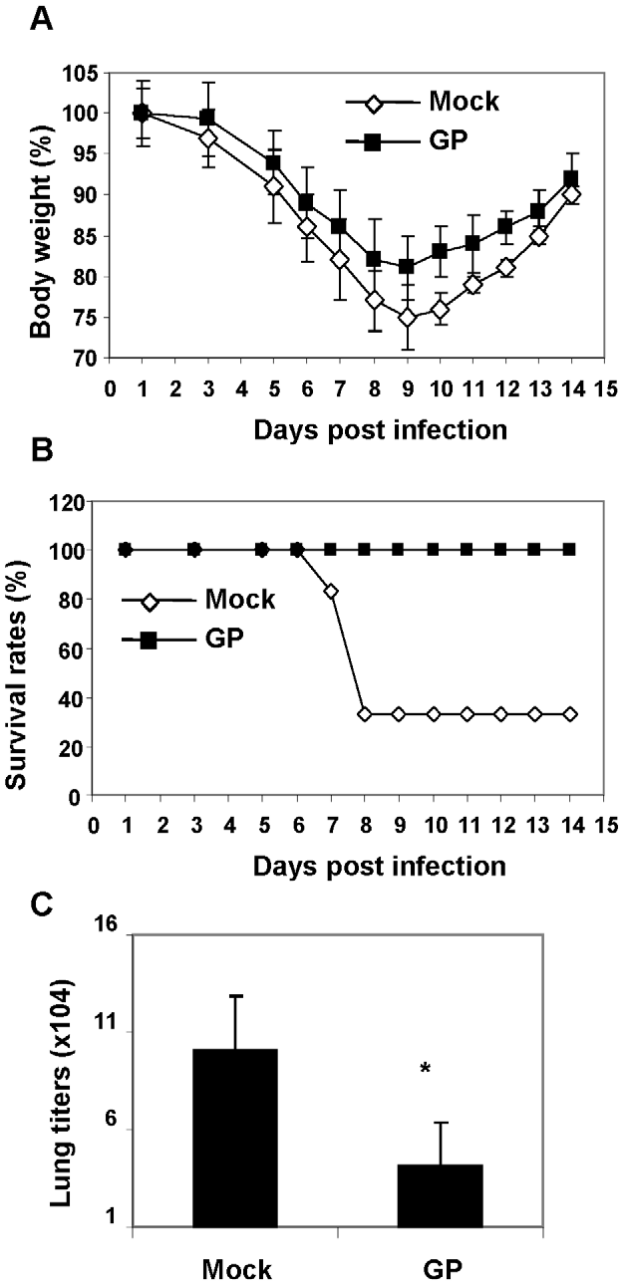
Ginseng polysaccharide pretreatment improved the survival after infection with H3N2 influenza virus. A) Changes in body weight (%). B) Survival rates (%). C) Lung viral titers at day 4 post infection (n = 4 out of 10 infected mice). BALB/c mice (n = 10 , 12 weeks old) were once pretreated intranasally with ginseng polysaccharide (25 mg/kg) 6 hours prior to infection with H3N2 subtype A/Philippines/82 virus (3 LD_50_). * p<0.05 between mock and GP groups.

To test if the protective effects of GP extended to a different subtype influenza virus, the A/Philippines/82 (H3N2 subtype) was used as a challenge virus (Fig. 3). The GP treated or naïve mice were challenged with a sub-lethal dose of A/Philippines/82 virus (2 LD_50_). Less body weight loss (15–20%) was observed in the GP treated compared to naïve mice that showed 25% average body weight loss (Fig. 3A). Also, the survival rates of GP-administered and naïve mice were 100% and 33% respectively (Fig. 3B). To better understand the effects of GP on improving protection, we determined viral loads at an early stage of infection and host responses in the surviving mice. 2.5 fold lower lung viral titers were observed in the GP-treated mice compared to the naïve control (Fig. 3C, p<0.05).

**Figure 4:**
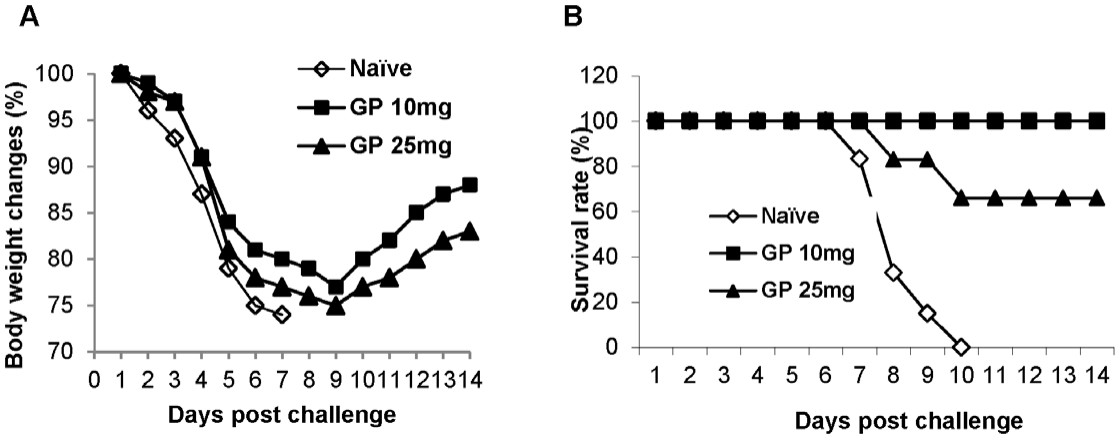
Intravenous pretreatment with ginseng polysaccharide provided protection against lethal infection with H3N2 influenza virus. A) Changes in body weight (%). B) Survival rates (%). Groups of mice (n = 5) intravenously administered ginseng polysaccharide (10 or 25 mg/kg) were infected with A/Philippines/82 virus (3 LD_50_), and survival rates monitored daily for 14 days. Mock: mice treated with PBS, GP 10 mg: mice treated with 10 mg/kg ginseng polysaccharide, GP 25 mg: mice treated with 25 mg/kg ginseng polysaccharide.

As an alternative route of delivery, different doses (10, 25 mg/kg) of GP were administered to mice intravenously 6 hr before infection with A/Philippines/82 virus (3 LD_50_). The low dose 10 mg/kg group showed 100% survival rate whereas a partial protection of 66% was observed in the 25 mg/kg group (Fig. 4). Nonetheless, the surviving mice in both groups showed severe body weight loss (Fig. 4A). All naïve mice died after infection with the same dose of A/Philippines/82 virus (3 LD_50_). Thus, GP might be partially effective in conferring protection through intravenous administration but high doses of GP would not enhance the survival protection. The results suggest that an optimal dose of GP administration confers the host with improved capacity to protect against lethal infection with H3N2 influenza strain.

### Ginseng polysaccharide contributes to protective immunity

**Figure 5:**
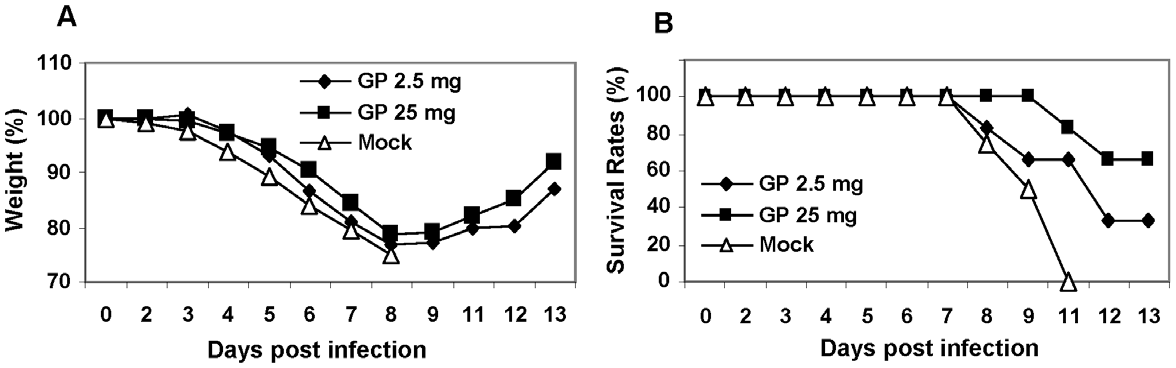
Ginseng polysaccharide effects on influenza infection. Mixtures of virus (A/California/2009, 3 LD_50_) and different amounts of ginseng polysaccharide (2.5 mg/kg, 25 mg/kg) were used to infect mice intranasally (n = 6). The body weight changes (A) and survival rates (B) were monitored daily for 14 days. Mock: naïve mice infected with virus in PBS, GP 2.5 mg: naïve mice infected with a mixture of virus and 2.5 mg/kg of ginseng polysaccharide, GP 25 mg: naïve mice infected with a mixture of virus and 25 mg/kg of ginseng polysaccharide.

To better understand the potential role of GP in conferring protection against influenza viral infection, we tested the direct effects of GP on viral infection in naïve mice. Mixtures of a lethal dose (3 LD_50_) of the 2009 H1N1 virus (A/California/04/2009) and GP were used to intranasally infect naïve mice. Naïve mice that were infected with a mixture of virus and PBS buffer showed severe body weight loss and all died (Fig. 5). In contrast, 2.5 mg/kg and 25 mg/kg GP provided 33% and 66% protection to naïve mice that were infected with a lethal dose of the 2009 H1N1 virus mixed with GP although the surviving mice showed a visible body weight loss similar to the infection in the PBS control (Mock). Therefore, these results suggest that GP may have a direct effect on improving the survival against influenza infection.

**Figure 6:**
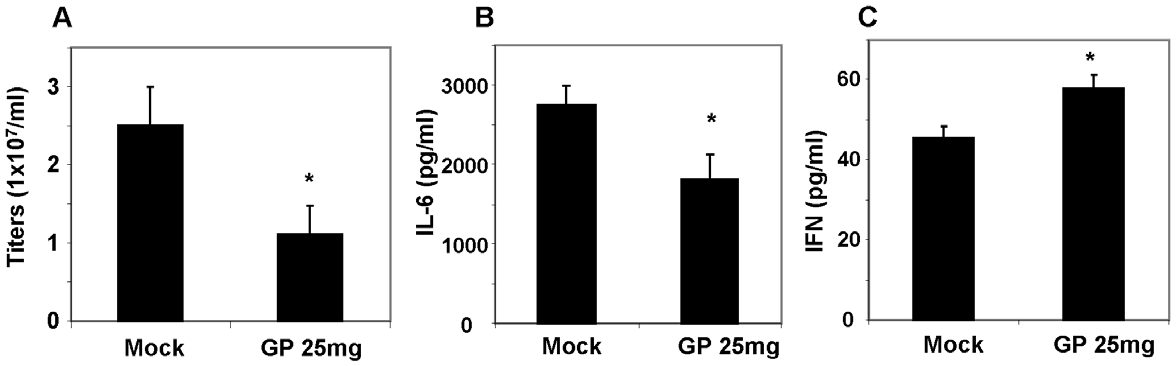
Lung viral titers and cytokines in mice infected with a mixture of ginseng polysaccharide and virus (A/California/2009, 3 LD_50_). Lung viral titers (A), interleukin (IL)-6 (B), and interferon (IFN)-γ levels (C) were determined at day 4 post infection with a mixture of ginseng polysaccharide and virus (A/California/2009, 3 LD_50_) (n = 5). Mock: naïve mice infected with virus in PBS, GP 25 mg: naïve mice infected with a mixture of virus and 25 mg/kg of ginseng polysaccharide. * p<0.05 between mock and GP 25 mg/kg groups as determined by Student's t-test.

Lungs were collected at day 4 post infection to determine lung viral titers and cytokine levels (Fig. 6). The GP treated group showed lower lung viral titers by 2.5 fold compared to that of the control group without GP (Fig. 6A). Also, the levels of inflammatory cytokine IL-6 was found to be lower in the GP treated group compared to the untreated control after lethal infection (Fig. 6B). In contrast, interferon (IFN)-γ was moderately higher in lungs of GP-treated mice compared to the infected naïve control mice (Fig. 6C). These results imply that GP-mediated protection might be partially due to inhibiting lung viral replication and inflammatory cytokines, and stimulating antiviral cytokine IFN-γ production.

### Ginseng polysaccharide improves cross protection by influenza vaccines

**Figure 7:**
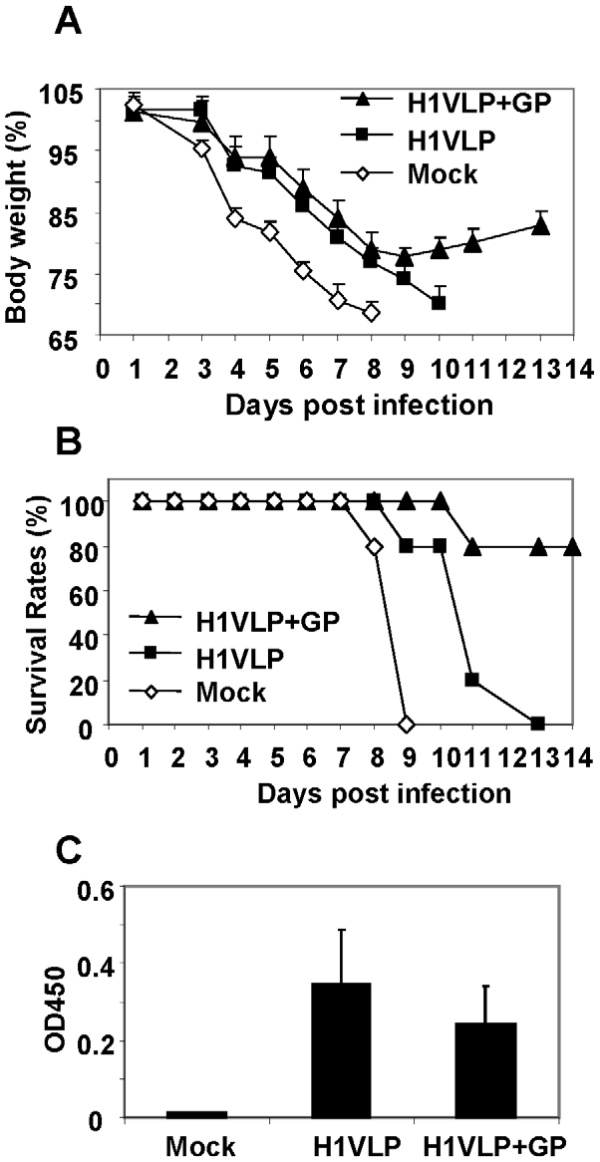
Ginseng polysaccharide enhances heterosubtypic cross protection in vaccinated mice. (A) Body weight changes. (B) Survival rates. Mice (n = 5) that were intranasally vaccinated with influenza VLPs (H1 VLP, 8 µg total protein) were intranasally treated with ginseng polysaccharide (5 mg/kg) daily for 12 days prior to infection with H3N2 heterosubtypic virus (A/Philippines/82, 6 LD_50_). (C) A/PR/8/34 virus specific serum antibody levels. Virus- specific antibody levels were determined in sera collected prior to challenge infection. H1 VLP: Vaccination with H1 VLPs, H1 VLP+GP: Daily treatment with GP for 12 days from the day 1 post vaccination with H1 VLPs, Mock: unvaccinated mice infected with virus.

Immunization with inactivated viral vaccine usually induces good protective immunity against the homologous virus infection but not against a heterosubtypic influenza A virus. Influenza virus-like particles (VLPs) have been suggested to be a promising candidate vaccine [18], [25]. Here, we determined the effects of GP pretreatment on induction of heterosubtypic protection in mice vaccinated with influenza VLPs containing HA and M1 derived from H1N1 A/PR8 virus (Fig. 7). Mice were intranasally immunized with influenza VLPs (8 µg total protein). From day 1 post vaccination, groups of naïve and vaccinated mice were treated daily with GP (5 mg/kg daily) via the intranasal route for 12 days. At day 13 post vaccination, mice were challenged with a lethal dose (6 LD_50_) of H3N2 subtype A/Philippines virus. All naïve mice became very sick, rapidly lost body weight below 75% and died or were euthanized. Also, all mice in the A/PR8 VLP vaccinated group died although the vaccinated mice showed a delay in mortality (Fig. 7A, 7B). The group of unvaccinated mice with GP treatment daily for 12 days exhibited severe loss in body weight similar to that of vaccinated mice without GP and showed 25% survival (data not shown). Importantly, 80% of mice that were vaccinated with A/PR8 VLPs and treated with GP daily for 12 days were survived lethal challenge with A/Philippines virus. Daily GP treatment did not increase the immune responses to the viral antigen (Fig. 7C). Also, daily treatment of a higher dose (25 mg/kg body) did not improve the cross protective efficacy (data not shown). These results suggest that daily GP treatment has the potential to improve the cross protective vaccine efficacy.

### Ginseng polysaccharide has antiviral activity

**Figure 8:**
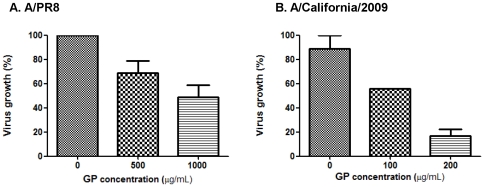
Effects of ginseng polysaccharide on the growth of influenza virus *in vitro*. A) Effects of ginseng polysaccharide on influenza A/PR8 virus. A diluted influenza A/PR8 virus (approximately 20,000 plaque forming units/ml) was mixed with different concentrations of GP (0, 500, 1000 µg/mL) and cultured for 3 days. B) Effects of ginseng polysaccharide on 2009 H1N1 pandemic influenza A/California/2009 virus. A diluted influenza A/California/2009 virus (approximately 4,000 plaque forming units/ml) was mixed with different concentrations of GP (0, 100, 200 µg/mL) and cultured for 5 days. The growth of viruses (A/PR8 virus, A/California/2009 virus) was determined by hemagglutination activity using chicken red blood cells, and presented as percentages compared to the controls (the wells without GP).

It would be of interest to determine whether GP treatment has effects on viral growth. When influenza A/PR8 virus was mixed with GP at different concentrations, the amounts of viral growth as measured by a hemagglutination activity assay were moderately lower up to 50% in a dose-dependent manner (Fig. 8A). High amounts of GP concentrations (500, 1000 µg/ml) were required to exhibit an inhibition effect on a high dose A/PR8 virus of approximately 20,000 plaque forming units (PFU) per milliliter. To determine the effects of GP on the growth of a different strain, the 2009 H1N1 pandemic virus (A/California/2009) was tested (Fig. 8B). The concentration of A/California/2009 virus was approximately 4000 PFU per milliliter and GP amounts in a range of up to 200 µg/ml were tested. Approximately five-fold lower levels of A/California/2009 virus production were observed at the GP concentration of 200 µg per milliliter (Fig. 8B). Also, a similar pattern of virus growth inhibition was obtained when mixtures of A/California/2009 virus and GP were used to infect eggs (data not shown). Therefore, these results suggest that GP may have the anti-viral growth activity against influenza viruses in a dose-dependent but strain-independent pattern.

## Discussion

Although GP has been known to have various immunomodulatory functions, its *in vivo* effects on viral infectious diseases are not very well studied. For the first time, we have investigated the effects of GP treatment on influenza virus infection in a mouse model. Intranasal or intravenous administration of mice with GP prior to infection conferred resistance to intranasal lethal infection by different influenza strains, resulting in increased survival rates but not preventing body weight loss. Also, naïve mice were partially protected when a lethal dose of influenza virus was mixed with GP prior to infection. Finally, intranasal pretreatment of vaccinated mice with GP improved their survival against heterosubtypic infection. Therefore, GP might have a preventive effect on influenza infection.

Intranasal administration with GP was tested in this study since influenza virus infects the host via the respiratory tract. The survival benefit against both H1N1 (A/PR8) and H3N2 (A/Philippines) viruses was observed with intranasal 25 mg/kg or intravenous 10 mg/kg doses. Higher doses of GP pretreatments did not increase the survival benefit. Although these dosage ranges used in our study were similar to those reported by a previous study demonstrating the beneficial effect of intravenous pretreatment with GP on decreasing septic complications by intraperitoneal infection with bacteria, variable optimum doses were previously reported depending on different septic models [16], [17]. Also, we found that timing of pretreatments influenced the efficacy of survival. Pretreatment more than one day earlier decreased the survival benefit and treatment post influenza infection did not improve protective efficacy (data not shown). This is consistent with previous findings that GP or ginseng treatment close to septic infection or after lipopolysaccharide stimulation was less or not effective [17], [26]. Further studies are needed to better understand the mechanisms by which pretreatments with GP exhibit survival benefit during influenza infection.

Pretreatment with GP resulted in lower lung viral titers than those observed in the untreated mice at day 4 post infection. This better control of lung viral replication observed was not because of increased anti-viral antibody immune responses in the GP treated group (data not shown). In support of this observation, beneficial effects of GP pre-treatment were also observed in B cell-deficient mice (data not shown). A possible mechanism is that GP may have anti-viral effects on influenza virus. Naïve mice infected with a mixture of a 2009 H1N1 influenza virus and GP showed improved survival compared to the control mice infected with the same dose of virus in PBS. Moderate decreases were observed in lung viral titers (approximately 2.5 fold) in the GP treated virus group. In this regard, it is interesting to note that the replication of influenza viruses mixed with GP was inhibited at moderate levels. However, the requirement of high concentration of GP indicates that the direct virucidal effects of GP are relatively weak and other additional mechanisms might be involved *in vivo*.

GP was also demonstrated to enhance the production of cytokines and reactive oxygen species by stimulating macrophages [12], [27]. In a recent study, GP was shown to stimulate dendritic cells resulting in enhanced production of IFN-γ [28]. In contrast, intravenous pretreatment of GP attenuated the production of serum pro- and anti-inflammatory cytokines after septic bacterial infection [16]. Infection of mice with a mixture of virus and GP resulted in higher levels of the antiviral cytokine IFN-γ and lower levels of proinflammatory cytokine IL-6 compared to those in the mice infected with virus only. Taken together, this study and others suggest a mechanism that GP might have antiviral activity against influenza virus by modulating host cytokines.

Infection with pathogenic influenza virus was shown to induce significant cell death probably via apoptosis causing tissue damages as a pathogenic mechanism of influenza [29]–[32]. Constitutive ERK1/2 activation is known to be important for the viability of malignant tumor/cancer cells and the phosphorylation of ERK1/2 plays a critical role in activating genes involved in cell cycle regulation and survival [33]–[35]. In preliminary studies, we found that influenza infection of human lung epithelial cell line (A549) induced significantly lower levels of ERK1/2 phosphorylation, indicating apoptosis-mediated cell death due to influenza virus infection. Examination of influenza infected cells under the microscope showed substantial cytopathic effects (data not shown). Importantly, pretreatment with GP resulted in preventing the down-regulation of ERK1/2 phosphorylation due to the subsequent infection with influenza virus. GP treatment of human lung epithelial cell line did not change the constitutive phosphorylation of ERK1/2 after infection with influenza virus (data not shown). Consistent with our observation, GP was reported to significantly increase the viability of peritoneal macrophage cells [36]. In addition, ginseng was shown to inhibit degradation of long-lived proteins and to stimulate protein synthesis similar to polypeptide growth factors [37]. Thus, it is speculated that maintaining the cell viability under the condition of viral infection-induced stress might be an another alternative mechanism for the protective effects by GP.

It is highly significant to enhance the cross protective efficacy of vaccination considering that current influenza vaccines induce strain-specific protection. This study first demonstrated that heterosubtypic protection of vaccinated mice was observed in mice received GP treatment daily although treatment of vaccinated mice with GP did not affect the host humoral immune responses to the vaccine antigen. Most people who consume ginseng are not immunologically naïve and are either vaccinated or immune to certain infectious agents because of natural exposure. It is also considered that immunomodulatory effects by GP treatments might play an important role in the outcomes of survival after influenza viral infection. Therefore, normal consumption of ginseng containing GP in healthy individuals would have beneficial effects on preventing unexpected influenza infections.
